# Clot lysis time and thrombin generation in patients undergoing transcatheter aortic valve implantation

**DOI:** 10.1007/s11239-024-03027-5

**Published:** 2024-08-08

**Authors:** Aleksander Siniarski, Aleksandra Gąsecka, Katarzyna Krysińska, Marta Frydrych, Jadwiga Nessler, Grzegorz Gajos

**Affiliations:** 1 Department of Coronary Artery Disease and Heart Failure, Faculty of Medicine, Institute of Cardiology, Jagiellonian University Medical College, Krakow, Poland; 2 St. John Paul II Hospital, Krakow, Poland; 3 1st Chair and Department of Cardiology, Medical University of Warsaw, Banacha 1aST, 02-097 Warsaw, Poland

**Keywords:** Fibrin clot, Thrombin generation, TAVI, Aortic stenosis

## Abstract

**Background:**

Aortic valve stenosis (AS) is the most prevalent valvular heart disease and is associated with a significant increase in mortality. AS has been shown to be linked with numerous coagulation system abnormalities, including increased fibrin deposition on the stenotic aortic valves. Transcatheter aortic valve implantation (TAVI) is the primary treatment method for patients at high surgical risk.

**Objectives:**

The aim of the study was to assess the impact of treating severe AS with TAVI on thrombin generation and clot lysis time (CLT).

**Methods:**

We studied 135 symptomatic AS patients recommended for TAVI by the local Heart Team. All measurements were performed before and 5-7 days after TAVI. Alongside clinical assessment and echocardiographic analysis, we assessed clot lysis time (CLT) and thrombin generation parameters, including lag time, peak thrombin generation, time to peak thrombin generation (ttPeak), and endogenous thrombin potential (ETP).

**Results:**

70 patients were included in the final analysis. After TAVI, there was a significant 9% reduction in CLT despite a 12% increase in fibrinogen concentration. We observed significant increase in lag time and ttPeak (20% and 12%, respectively), and 13% decrease in peak thrombin concentration compared to pre-procedural levels. Multivariable linear regression analysis demonstrated that baseline CLT and C-reactive protein (CRP) levels were independent predictors of significant reduction in mean aortic gradient, defined as TAVI procedure success.

**Conclusions:**

CLT and peak thrombin concentration decreased, while Lag time and ttPeak increased significantly after TAVI. Multivariable linear regression analysis demonstrated CLT and CRP levels as independent predictors of achieving a reduction in mean aortic gradient, defining TAVI procedure success.

**Graphical abstract:**

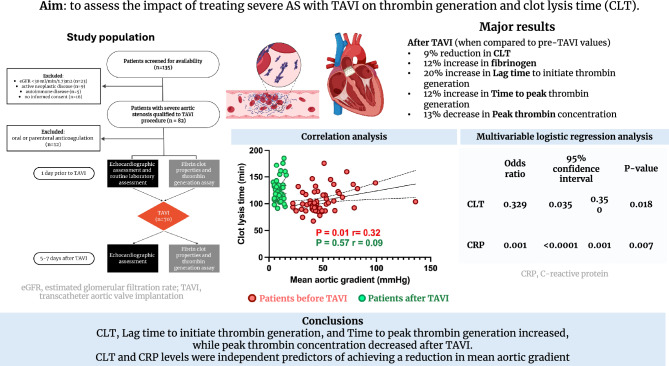

**Supplementary Information:**

The online version contains supplementary material available at 10.1007/s11239-024-03027-5.

## Highlights

- Treating severe aortic valve stenosis (AS) with transcatheter aortic valve implantation (TAVI) significantly reduced clot lysis time (CLT) by 9% despite a 12% increase in fibrinogen concentration.
- After TAVI, there was a notable increase in lag time and time to peak thrombin generation (ttPeak) by 20% and 12%, respectively, along with a 13% decrease in peak thrombin concentration.
- Multivariable linear regression model identified baseline CLT and C-reactive protein (CRP) levels as independent predictors of a reduction in mean aortic gradient.
- The findings suggest that TAVI not only alleviates aortic valve stenosis but also favorably modifies coagulation parameters, highlighting the potential role of CLT and CRP levels in predicting procedural outcomes.

## Introduction

Aortic stenosis (AS) has become the predominant form of primary heart valve disease and a significant contributor to cardiovascular (CV) morbidity and mortality [1]. Echocardiography stands as the cornerstone for diagnosing and assessing AS, serving as the principal non-invasive imaging modality for evaluating this condition [1]. AS incidence is escalating mainly due to the advancing age of the population, where the prevalence of 3.5% in those above 75 years of age [2]. Severe AS required urgent surgical or interventional treatment, as more than 50% of patients with AS die within 2 years after the symptoms appear, if treated conservatively [3]. Surgical aortic valve replacement (SAVR) is considered the standard treatment for patients with AS who are at low perioperative risk. However, it may not be suitable for elderly patients or those at high perioperative risk due to comorbidities. In contrast, transcatheter aortic valve implantation (TAVI) has emerged as a less invasive alternative to SAVR [4]. Initially reserved for elderly patients, the utilization of TAVI has recently expanded to younger patients with AS who are at intermediate or even low perioperative risk [5, 6].

Patients with AS present with numerous blood clot abnormalities [7–10]. For example, there is an association between the thickness of fibrin layer within the aortic valve and maximal and mean aortic gradients in AS patients [8]. Fibrin deposition, predominantly co-localized with the tissue factor, was observed within the valve leaflets of individuals with advanced AS [8]. Additionally, thrombin generation and fibrin clot formation correlated with the degree of fibrin accumulation within the leaflets, suggesting a potential contribution to the progression of AS [8]. Furthermore, it was demonstrated that individuals scheduled for TAVI exhibit more prothrombotic fibrin clot phenotype, featuring a denser fibrin meshwork and greater resistance to lysis compared to those undergoing SAVR [11]. This observation may partially account for the increased thromboembolic risk following TAVI [12–14]. This phenomenon was further illustrated by the increased levels of thrombin-antithrombin complexes and prothrombin fragments following TAVI compared to percutaneous coronary intervention for acute myocardial infarction [15]. After AS treatment with both SAVR or TAVI procedure, less turbulent blood flow is restored [16, 17], which might result in the reversal of the prothrombotic state [18]. Finally, TAVI itself might also induce a hypercoagulable state due to peri-procedural platelet activation, resulting from the injured endothelial surface, which causes thrombocytosis [19].

We hypothesized that the restoration of laminar flow after TAVI is reflected by a decrease in blood coagulation and lysis parameters. To assess the impact of restoring low-gradient, laminar blood flow through the implanted bioprosthetic valve on thrombin generation and clot lysis, we compared thrombin generation and fibrin clot lysis parameters before and after TAVI.

## Methods

### Study design

This prospective study was performed at 1st Chair and Department of Cardiology, Medical University of Warsaw, Poland in collaboration with Department of Coronary Artery Disease and Heart Failure, Institute of Cardiology, Jagiellonian University Medical College at St. John Paul II Hospital, Kraków, Poland. A complete assessment of thrombin generation and clot lysis were conducted in the Krakow Center for Medical Research and Technologies at the St. John Paul II Hospital, Kraków.

The design and protocol of the study was compliant with Declaration of Helsinki and was approved by Ethics Committee of the Medical University of Warsaw (Approval Number: KB/128/2018, KB/4/A2021). Informed consent was obtained from all study participants.

### Study participants

We enrolled 135 patients with advanced symptomatic AS who were qualified for elective TAVI as per local Heart Team recommendation. Severe AS was defined as aortic valve area (AVA) < 1.0 cm^2^ or an indexed AVA < 0.6 cm^2^/m^2^, calculated using the continuity equation on transthoracic echocardiography (TTE) [1, 4]. The indication for TAVI was determined in accordance with the current guidelines of the European Society of Cardiology (ESC) [4]. It was determined that patients qualified for TAVI had an elevated surgical risk, defined as at least 4% in EuroScore II or STS calculator [4]. To distinguish between severe and pseudo-severe AS, the following tests were additionally performed if needed: (1) dobutamine stress echocardiography in low-flow, low-gradient, reduced left ventricular ejection fraction (LVEF) patients and (2) computed tomography with aortic valve calcium score assessment in low-flow, low-gradient, preserved LVEF patients. Inclusion and exclusion criteria are listed in Table 1.

**Table 1:** Inclusion and exclusion criteria in the study

Inclusion criteria	Exclusion criteria
Age ≥ 18 years AVA < 1,0 cm^2^ or AVAi < 0,6 cm^2^/m^2^ calculated with use of continuity equation in TTE Technical eligibility for TAVI	Oral or parenteral anticoagulation Chronic kidney disease (glomerular filtration rate < 30 mL/min/1,73m^2^) Active neoplastic disease Autoimmune diseases Pregnancy or breastfeeding Valve-in-valve implantation Coagulation disorders Active bleeding History of bleeding disorder Severe thrombocytopenia (platelet count < 50,000/µL) Severe liver insufficiency (Child–Pugh score in class C)

*AVA* aortic valve area, *AVAi* AVA indexed for body surface area, *TAVI* transcatheter aortic valve implantation, *TTE* transthoracic echocardiography

### TAVI procedure

A multidetector computed tomography angiography (CTA) was used to select the size and approach for transcatheter aortic valves. TAVI procedures were performed using femoral access in a hybrid operating room. The procedure was performed by an interventional cardiologist in collaboration with a cardiac surgeon. Following TAVI, single antiplatelet therapy (mostly acetylsalicylic acid) was administered in patients with no indication for oral anticoagulation (OAC) [1]. If the patient had indications before TAVI to take dual antiplatelet therapy (DAPT), such therapy was maintained as recommended.

### Sample collection and routine laboratory tests

During hospitalization, blood samples were collected at two time points: (1) one day before the TAVI procedure, and (2) 5–7 days after (at hospital discharge). A fasting blood sample of 25 ml was collected from the antecubital vein and immediately preserved in tubes containing 3.2 trisodium citrate. Sample processing took place within 60 min of blood collection.

The Cobas 8000 CC/c702 + c502 Roche and Dimension EXL Siemens was used to analyze serum levels of total cholesterol, low-density lipoprotein cholesterol (LDL-C), high-density lipoprotein cholesterol (HDL-C), triglycerides, glucose, glycated hemoglobin (HbA1c), and high sensitivity C-reactive protein (hsCRP). Creatinine levels were determined and estimated glomerular filtration rate (eGFR) calculated using the chronic kidney disease epidemiology collaboration (CKD-EPI) formula. A complete blood count (CBC), including red and white blood cell count, hemoglobin, hematocrit, red cell distribution width, platelet distribution width, and platelet count, was assessed with Yumizen H 2500 Horiba and XN2000 Sysmex.

### Laboratory investigations of coagulation parameters

Venous blood was obtained from the antecubital vein between 7:00 and 09:00 AM using citrated tubes (9:1 ratio of 0.106 M sodium citrate; Monovette, Sarstedt, Nümbrecht, Germany). Subsequently, the collected blood underwent centrifugation at 2500 g and 20 °C for 20 min to obtain platelet-poor plasma. This plasma was rapidly snap-frozen within 30 min of collection and stored in aliquots at − 80 °C until analysis.

#### Thrombin generation

Thrombin generation kinetics were assessed using the Calibrated Automated Thrombogram (CAT, Thrombinoscope BV, Maastricht, the Netherlands) following the manufacturer’s instructions. The measurements were conducted in a 96-well plate fluorometer (Ascent Reader, Thermolabsystems OY, Helsinki, Finland) equipped with the 390/460 filter set at a temperature of 37 °C. In summary, 80 µL of platelet-poor plasma were diluted with 20 µL of a reagent containing 5 pM recombinant tissue factor (TF), 4 micro-molar phosphatidylserine/phosphatidylcholine/phosphatidylethanolamine vesicles, and 20 µL of FluCa solution (HEPES, pH 7.35, 100 nM CaCl2, 60 mg/mL bovine albumin, and 2.5 mM Z-Gly-Gly-Arg-7-amino-4-methylcoumarin). The Peak thrombin represents the maximum concentration of thrombin formed during the recording time, while the area under the curve corresponds to the endogenous thrombin potential (ETP). Lag time demonstrates the initiation phase of coagulation, and time to peak (ttPeak) signifies the propagation phase of thrombin generation. Each plasma sample underwent duplicate analysis, and the intraassay variability was determined to be 7%.

#### Plasma clot lysis assay

The fibrinolysis capacity was assessed utilizing a clot lysis time (CLT) assay recommended by the International Society on Thrombosis and Haemostasis (ISTH) Subcommittee, as detailed previously [20]. In brief, citrated plasma was combined with 15 mM calcium chloride, 0.5 U/mL human thrombin (Merck), 15 µM phospholipid vesicles (Rossix, Mölndal, Sweden), and 18 ng/mL recombinant tissue plasminogen activator (rtPA, Boehringer Ingelheim, Germany). Subsequently, the mixture was transferred to a microtiter plate, and its turbidity was measured at 405 nm at a temperature of 37 °C. CLT is defined as the time interval between the midpoints of the clear-to-maximum-turbid transition and the midpoints of the maximum-turbid-to-50% clear transition (50% lysis). Lysis variables had intraassay coefficients below 8%.

### Echocardiographic evaluation

Full echocardiographic evaluation was performed in all studied subjects. Precise measurements of aortic valve including maximal velocity through aortic valve (Vmax), aortic valve area (AVA), aortic valve area indexed for body surface area (AVAi), maximal and mean aortic gradient, left ventricular ejection fraction. The examination was performed be an experienced cardiologist. All scans were performed with a Philips Sparq ultrasound machine (Philips Healthcare, Andover, MA) using a phased-array transducer probe (2–4 MHz), with additional analysis using Philips Q-Station 3.3.2.

### Endpoints

The primary endpoint was the change in (1) fibrin clot properties, and (2) thrombin generation before vs. after TAVI. The secondary endpoint was the predictive value of baseline (1) thrombin generation and (2) clot lysis time for changes in aortic valve flow parameters, namely: AVA, AVAi, maximal or mean aortic gradient.

### Statistical analysis

Since there are no data regarding the differences in thrombin generation, fibrin clot properties and clot susceptibility to lysis before and after TAVI, power calculation for the primary endpoint was based on the differences in fibrin clot properties in patients scheduled for TAVI, compared to those undergoing SAVR [21]. Patients scheduled for TAVI had, on average, a 0.1-fold higher clot density, compared to SAVR patients. The required sample size was calculated by a 2-sided t-test at a significance level of 0.05 with the following assumptions: (i) mean difference between the groups = 1.0, (ii) standard deviation (SD) ± 2.0, and (iii) nominal test power = 0.8. It was estimated that a total of 64 patients should be enrolled in the study to observe a difference in clotting parameters before and after TAVI.

Categorical variables are presented as number and percent. The Shapiro–Wilk test was used to assess normal distribution of continuous variables. Continuous variables were presented as mean and SD or median with interquartile range (IQR). Spearman rank correlation coefficients were calculated for associations between continuous variables with non-normal distributions. Univariable linear regression analyses demonstrated relationships between clinical variables and blood coagulation variables. Paired t-test of Wilcoxon signed rank test was used for repeated measures analysis, between two analysed timepoints. A 2-sided p-value below 0.05 was considered significant. Statistical analysis was conducted using GraphPad Prism version 8.0.1 (San Diego, CA, USA) and IBM SPSS ver. 28.0 (Armonk, NY, USA).

## Results

**Fig. 1:**
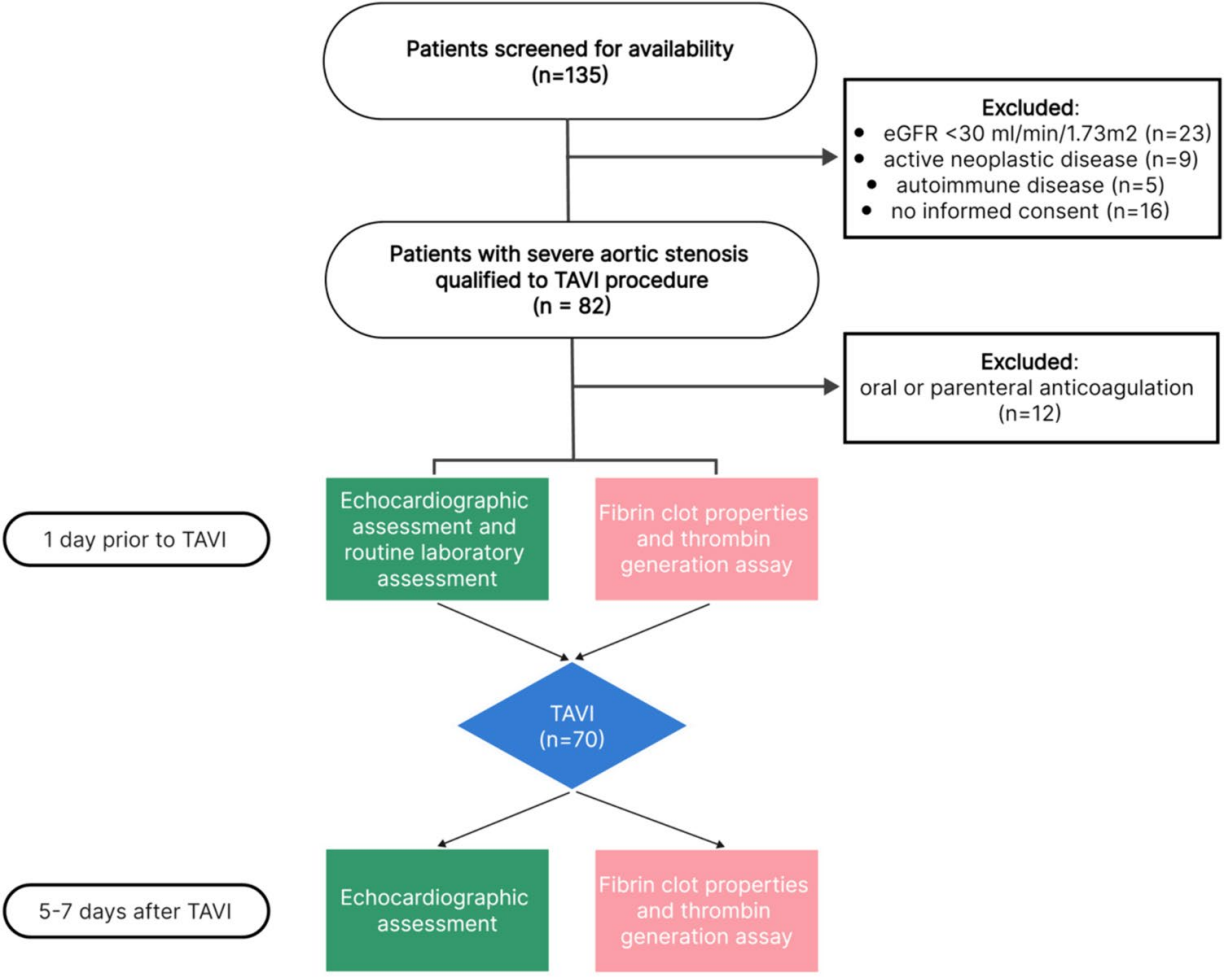
Flow chart of the study. *TAVI* transcatheter aortic valve implantation. Data demonstrated as number in brackets in each point of the study

Out of the 135 patients who underwent TAVI between November 2018 and June 2020, 70 patients were included in the final analysis. The complete flowchart of the study is demonstrated in Fig. 1. Full baseline characteristics of the study population is shown in Table 2. The mean age was 78 years (± 7.3 years) and 47% were women. Patients presented with multiple comorbidities, including type 2 diabetes (34.8) arterial hypertension (86.4%), chronic kidney disease (16.7%), chronic obstructive pulmonary disease (12.1%).

**Table 2:** Baseline characteristics of patients

Total number of patients n = 70		
Age (years)	80.0 (74.0–84.0)	
Gender, male	31 (46.97%)	
BMI [kg/m2]	28.5 ± 4.8	
Medical history and concomitant diseases1		
NYHA III/IV	23 (34.8%)	
EuroSCORE II [%]	3.70 (2.12–4.77)	
Prior myocardial infarction	14 (21.21%)	
Prior PCI	29 (43.94%)	
Prior CABG	3 (4.5%)	
Atrial fibrillation	4 (6.1%)	
Prior stroke/TIA	7 (10.6%)	
CKD > G3a	11 (16.7%)	
Diabetes mellitus	23 (34.8%)	
Hypertension	57 (86.4%)	
COPD	8 (12.1%)	
Laboratory data		
Hemoglobin [g/dL]	11.4 ± 2.4	
Leukocytes [G/l]	7.24 (6.59–8.49)	
Thrombocytes [G/l]	185 (154–225)	
Creatinine [mg/dL]	1.07 (0.89–1.32)	
Estimated GFR [ml/min/1.73 m^2^]	57.0 (46.0–70.0)	
NT – proBNP [ng/l]	973 (423–2727)	
CRP [mg/l]	1.20 (0.39–4.48)	
Echocardiographic assessment before TAVI		
Ejection fraction [%]	60.0 (50.0–62.0)	
V max [m/s]	4.20 (4.05–4.50)	
Gradient max	72.0 (65.5–85.0)	
Gradient mean	43.0 (40.0–53.0)	
AVA (VTI)	0.80 (0.67–0.87)	
AVAi	0.44 (0.36–0.49)	
Low-flow, low-gradient AS	10 (16.39%)	
Procedural characteristics		
Access site	Transfemoral	65 (100%)
Prothesis size [mm]	23	3 (4.62%)
	25	13 (20.0%)
	26	6 (9.23%)
	27	14 (21.5%)
	29	13 (20.0%)
	30	1 (1.54%)
	34	15 (23.1%)
Valve type	Portico	19 (29.2%)
	Evolut R	18 (27.7%)
	Evolut PRO	5 (7.69%)
	Acurate Neo2	12 (18.5%)
	Acurate Neo	4 (6.15%)
	CoreValve Evolute R	5 (7.69%)
	Hydra	1 (1.54%)
	Navitor	1 (1.54%)
Echocardiographic assessment after TAVI		
Ejection fraction [%]	60.0 (54.8–65.0)	
V max [m/s]	2.20 (1.80–2.48)	
Gradient max	19.0 (14.0–22.8)	
Gradient mean	10.0 (7.00–11.0)	
AVA (VTI)	1.91 (1.59–2.20)	
AVAi	1.07 (0.97–1.29)	
Paravalvular leak (type 3 or 4)	0 (0.0%)	
Procedure complications		
Bleeding requiring packed red blood cells transfusion	5 (7.81%)	
Surgical intervention within the puncture site	4 (6.25%)	
Valve thrombosis	0 (0.0%)	
Valve re-intervention	0 (0.0%)	
New pacemaker implantation	1 (1.64%)	
Death form any cause	2 (3.28%)	
Death from cardiovascular causes	3 (4.92%)	
Myocardial infarction	3 (4.92%)	
Stroke/TIA	0 (0.0%)	
Post-TAVI procedure concomitant medications		
Acetylsalicylic acid	59 (95.2%)	
P2Y12 inhibitor	55 (88.7%))	
Anticoagulant	0 (0%)	
Beta-blockers	50 (80.65%)	
ACE inhibitors	44 (71%)	
Sacubitril/valsartan	1 (1.61%)	
MRA	15 (24.6%)	
Diuretics	50 (80.6%)	
Statins	53 (85.5%)	
Proton pump inhibitors	44 (71%)	
Type 2 diabetes medications		22 (35.5%)
	Metformin	13 (21%)
	Sulfonylureas	5 (8.06%)
	Insulin	9 (14.5%)
	Acarbose	1 (1.61%)

Data presented as median (interquartile range) or mean ± standard deviation

*AVAi* indexed Aortic Valve Area, *BMI* Body Max Index, *CABG* coronary artery bypass graft, *CKD* chronic kidney disease, *COPD* chronic obstructive pulmonary disease, *CRP* C-reactive protein, *AVA (VTI)* aortic valve area (velocity time integral), *GFR* glomerular filtration rate, *MRA* mineralocorticoid receptor antagonist, *NT-proBNP* N-terminal pro-B-type natriuretic peptide, *PCI* percutaneous intervention, *TIA* transient ischemic attack

### Fibrin clot formation properties before and after TAVI

**Fig. 2:**
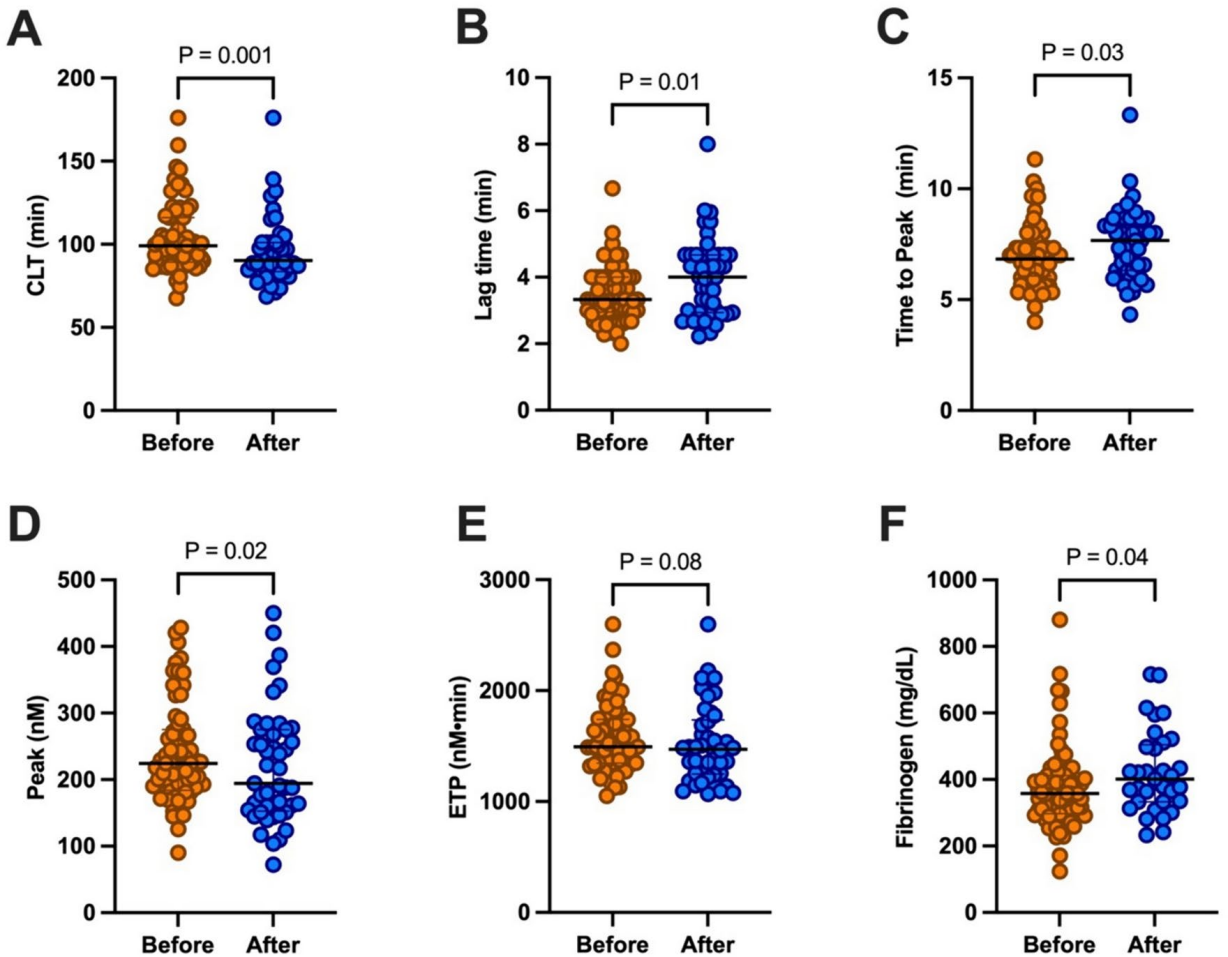
Comparison of properties of fibrin clot formation before and after TAVI. Data shown as median and interquartile range (horizontal line and whiskers, respectively). *CLT* clot lysis time, *ETP* endogenous thrombin potential, *Lag time* time to initiate thrombin generation, *Peak* peak thrombin concentration, *Time to peak* time to peak thrombin concentration

Following TAVI, a significant 9% reduction in CLT was observed, despite a concurrent 12% increase in fibrinogen concentration. Significant alterations in thrombin generation parameters were also noted, with a 20% increase in Lag time and a 12% increase in Time to peak. Moreover, there was a significant decrease (− 13%) in Peak thrombin concentration post-procedure compared to pre-procedural levels. Notably, no discernible differences were observed in ETP. Detailed comparisons of fibrin clot lysis time and thrombin generation before and after TAVI can be found in Fig. 2 and Table 3.

**Table 3:** Fibrin clot properties before and after TAVI

	Before TAVI	After TAVI
CLT (min)	99 (89.3–116)	90.3 (83.6–101)
Lag time (min)	3.33 (2.96–4.00)	4.00 (2.94–4.67)
Time to Peak (min)	6.84 (6.07–7.59)	7.67 (6.32–8.41)
Peak (nM)	224 (184–275)	194 (153–275)
ETP (nM x min)	1494 (1347–1742)	1472 (1249–1736)
Fibrinogen (mg/dL)	358 (297–412)	401 (333–505)

Data presented as median (interquartile range)

*CLT* clot lysis time, *ETP* endogenous thrombin potential, *Lag time* time needed to initiate clotting, *Peak* peak thrombin concentration, *TAVI* transcatheter aortic valve implantation, *Time to Peak* time to peak thrombin concentration

### Association between clot lysis time, thrombin generation and echocardiographic variables

**Fig. 3:**
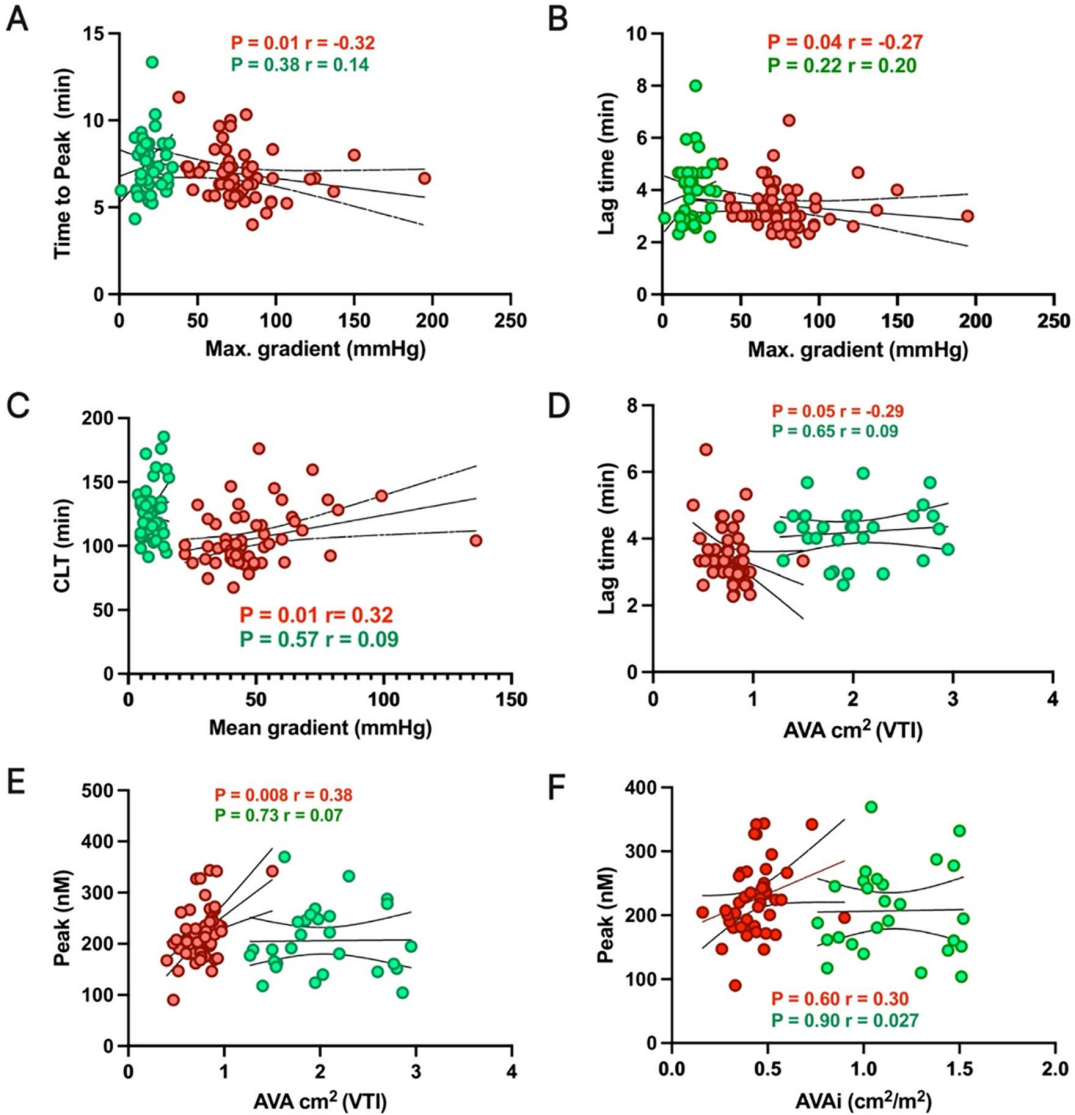
Correlations between selected fibrin clot, thrombin generation parameters and echocardiography parameters before versus after TAVI. The data are presented as Spearman correlations of the analyzed variables. Correlations of analyzed variables before the TAVI procedure are demonstrated in red, while those after TAVI are shown in green. P-values and correlation coefficients ® are provided in each panel of the figure. Here we demonstrated, the correlations between time to peak thrombin generation and maximal aortic gradient (Panel A); Lag time and maximal aortic gradient (Panel B); CLT and mean aortic gradient (Panel C); Lag time and AVA (Panel D); peak thrombin generation and AVA (Panel E), and non-significant correlations between peak thrombin generation and AVAi. *AVA* aortic valve area, *AVAi* AVA indexed for body surface area, *Max. gradient* maximal aortic gradient, *Lag time* time to initiate thrombin generation, *Peak* peak thrombin generation, *Time to peak* time to reach peak thrombin generation, *CLT* clot lysis time

We investigated the correlation between fibrin clot properties, thrombin generation and echocardiographic parameters both before and after TAVI procedure. Before TAVI we found significant correlations between time to peak thrombin generation, lag time and maximal aortic gradient (p = 0.01 and p = 0.04; Fig. 3A, B, respectively). Furthermore, we observed significant correlation between mean aortic gradient, AVA and CLT, but not between AVA and the lag time (p = 0.01 and p = 0.049; Fig. 3C, D, respectively). We also observed a significant association between peak thrombin and time to peak thrombin concentration and AVA. However, both variables when indexed for body surface area (AVAi), those correlations lost significance (p = 0.008, p = 0.60 and p = 0.02, p = 0.08 respectively). Importantly, all post-TAVI correlations were not associated with any aortic valve parameters or EF evaluations. All performed correlations between CLT and thrombin generation assay were demonstrated in Supplementary materials (Tables S1 and Table S2).

### Univariate and multivariate analysis

Independent predictors of decrease of mean aortic gradient, which is defined as procedure success are presented in Table 4. Full univariable and multivariable analysis is presented in the supplementary Table 3. The multivariable logistic regression model demonstrated that CLT and CRP were independently associated with TAVI procedure success, defined as a decrease in mean aortic gradient (Table 4).

**Table 4:** Multivariable logistic regression model for prediction of a decrease (delta) in mean aortic flow gradient, representing hemodynamic success of TAVI

Variable	OR	95% CI		p-value
		Lower	Upper	
CLT	**0.329**	**0.035**	**0.350**	**0.018**
Prior MI	− 0.054	− 10.972	7.187	0.674
Hemoglobin	− 0.066	− 2.381	1.405	0.603
CRP	**0.416**	**0.000**	**0.001**	**0.007**
Vmax	0.252	− 0.933	11.880	0.091

*OR* 0.001 (95% CI <0.0001 - 0.001)

Only variables predictive of the outcome in univariable analysis are presented in the Table. Full results of univariable analysis are available in the Supplementary Table S3. *95% CI* 95% confidence interval, *CLT* clot lysis time, *CRP* C-reactive protein, *MI* myocardial infarction; *OR*, odds ratio, *Vmax* maximal blood velocity measured through aortic valve

## Discussion

To the best of our knowledge, this study represents the first investigation to compare CLT and thrombin generation before and after TAVI. Our study cohort comprised elderly individuals with a very-high cardiovascular risk profile, including a majority exhibiting high-flow, high-gradient severe aortic stenosis (AS) and elevated surgical risk. The findings of this study clearly show that despite an increase in fibrinogen concentration, CLT decreased following TAVI, indicating a reduction in fibrin clot resistance to lysis. Additionally, patients after TAVI exhibited higher Lag time, prolonged time to peak thrombin generation, and reduced peak thrombin concentration compared to their pre-procedural values, indicative of a more favorable, less prothrombotic plasma clot phenotype. Moreover, our analysis revealed significant associations between pre-TAVI parameters such as CLT, time to peak thrombin, and peak thrombin concentration with echocardiographic markers of AS severity. Finally, we demonstrated that CLT was an independent predictor of significant decrease in mean aortic gradient, confirming successful TAVI procedure.

The mechanistic explanation of increased thrombosis risk in patients with AS is complexed. In patients with advanced AS, significant amounts of fibrin, often co-localized with tissue factor (TF), were found within valve leaflets, potentially contributing to AS progression [8]. Moreover, thrombin generation and fibrin clot formation correlate with the extent of fibronectin presence within leaflets, indicating a potential role in disease advancement [8]. Additionally, detectable TF activity in severe AS patients was associated with higher levels of prothrombin fragment 1.2, suggesting increased thrombin generation [22]; what was similarly observed in our study. Accumulation of lipoproteins has been observed in stenotic aortic valves, correlating with the development of this valvular disorder. Furthermore, lipoprotein(a) (Lp[a]) served as the primary carrier of oxidized phospholipids, posing a prothrombotic risk and contributing to valve stenosis progression [23–25]. Recent study also revealed that elevated levels of oxidized phospholipids (OxPLs) in severe AS patients were linked with impaired fibrinolysis, prolonged clot lysis time (CLT), and disease severity, with increased OxPL expression in aortic valves of patients with elevated serum lipoprotein(a) [Lp(a)] concentrations [24]. Furthermore, the presence of neutrophil extracellular traps (NETs) in stenotic valves and their association with AS severity suggest novel mechanisms contributing to disease progression [26].

Moreover, assessment of fibrin clot properties and thrombin generation were found to be important predictors of various cardiovascular diseases [27, 28]. It has been investigated in acute myocardial infarction, chronic coronary syndrome, and heart failure [29–33]. Recently, in a large randomized controlled trial derived data, it was found that CLT was an independent predictor of cardiovascular outcomes following acute myocardial infarction [34].

Other biomarkers of hypercoagulable state were also investigated in AS patients. A significant association was demonstrated between fibrin layer thickness in AS valves and aortic gradients [8]. Notably, advanced AS was marked by fibrin deposition, linked with tissue factor [8]. Thrombin generation and clot formation was correlated with fibrin accumulation, potentially influencing AS progression [8]. Moreover, patients undergoing TAVI showed more prothrombotic fibrin clot phenotype compared to SAVR patients [11], potentially contributing to increased thromboembolic risk [12–14]. In a study conducted by Jaworska-Wilczynska et al., peak thrombin concentration was found to have a negative correlation with peak aortic valve velocity, while it exhibited a positive association with lag time [11]. Additionally, it was observed that lag time was negatively associated with AVA [11]. These findings align with our results, which demonstrated a positive association between peak thrombin concentration and AVA, as well as maximal aortic gradient, while showing a negative association with time to peak thrombin concentration. Interestingly, the correlations we demonstrated before the procedure almost completely disappear after performing the TAVI procedure, which is a novel observation. However, lag time showed an opposing correlation, likely due to heterogeneity in group representation and various cardiovascular risk profile differences between patients treated with TAVI or SAVR [11].

Additionally, elevated thrombin-antithrombin complexes and prothrombin fragments were found in patients following TAVI procedure [15]. Regardless of the type of intervention performed on the stenotic aortic valve, treatment of AS with SAVR or TAVI restores laminar flow, potentially reversing the prothrombotic state [18]. On the other hand, TAVI procedure was shown to induce hypercoagulable state via platelet activation [19]. In our study, we observed a substantial decrease in CLT following the procedure, despite the initial rise in fibrinogen levels typically associated with the procedure itself [35]. Furthermore, favorable changes were noted not only in lag time but also in time to peak and peak thrombin concentration following TAVI in our cohort.

Dimberg et al. demonstrated that preoperative plasma thrombin potential is elevated in patients with isolated AS compared to those with coronary artery disease, whereas thromboelastometry did not differ between these patient cohorts [36]. Of note, preoperative bleeding assessment was not significantly associated with plasma thrombin potential and only to a limited extent with thromboelastometry in patients undergoing elective cardiac surgery for both AS and coronary artery disease [36]. In our study, the ETP showed no significant association with echocardiographic parameters confirming the severity of AS, nor did it significantly differ in patients post-TAVI compared to pre-procedural values. However, comparing patients treated with SAVR to those with coronary artery disease involves analyzing different interventions (low vs. high invasive) and clinical profiles. Moreover, this comparison was made against a distinct group of patients with very high cardiovascular risk, what unbales reliable comparison of those cohorts.

The study by Hang Chi et al. aimed to examine the levels of plasma extracellular vesicles (EVs) and their potential role in inducing procoagulant activity among patients undergoing TAVI alone or in combination with percutaneous coronary intervention (PCI) [37]. This study demonstrated the association between plasma EVs levels and hypercoagulability in patients following TAVI, particularly in those undergoing TAVI and PCI combined [37]. It was demonstrated that certain MicroRNAs (miRNAs) were associated with platelet function [38–40] and acute myocardial infarction patients (AMI) [41], suggesting the potential utility of these miRNAs as novel biomarkers for predicting major adverse cardiovascular and cerebrovascular events (MACCE) following TAVI. Eyileten et al., revealed that the expression levels of miR-223 and miR-125b increased post-TAVI compared to baseline measurements [18]. Furthermore, the authors demonstrated that a low baseline expression of miR-223 was significantly associated with nearly a three-fold increase in MACCE in patients with severe aortic stenosis scheduled for TAVI [18]. In our investigation, after a multivariable logistic regression analysis, we established that CLT emerged as an independent predictor of the TAVI procedure success defined as a reduction in the mean aortic gradient.

## Limitations

This study has several limitations that should be noted. Firstly, the number of participants is limited; however, we conducted a power sample calculation to ensure the validity of our results. Secondly, we only collected samples at two time points: before the TAVI procedure and shortly after. This means we lack information on how the analyzed parameters change over a longer clinical follow-up, including the occurrence of MACCE after TAVI. Thirdly, the observational nature of this study allows us to draw suggestions from the analyzed relationships; however, it should not lead to conclusions about the causality of observed phenomena. Ultimately, this translates into data that generate hypotheses for further research. Moreover, a subanalysis by valve type (which could have impacted the study results) was not performed due to a lack of statistical power. Additionally, we acknowledge the relatively high in-hospital death rate, which we hypothesize may be due to the advanced age (median 80 years) and high comorbidity rate of the patients; however, specific causes of death were not collected beyond categorization into cardiovascular and non-cardiovascular causes. Finally, while following the same patients over time helps minimize the influence of confounding factors, we cannot rule out the possibility of unknown factors affecting our results.

## Conclusions

We showed that despite increased fibrinogen concentration, CLT decreased post-TAVI, indicating increased susceptibility to lysis. Post-TAVI patients showed prolonged lag time, and time to peak thrombin generation, but also reduced peak thrombin concentration, indicative of a less prothrombotic plasma clot phenotype. Additionally, pre-TAVI parameters such as CLT, time to peak thrombin, and peak thrombin concentration correlated significantly with echocardiographic markers of AS severity. Notably, CLT emerged as an independent predictor of significant post-TAVI decrease in mean aortic gradient, what was not previously published.

## Supplementary Information

Below is the link to the electronic supplementary material.

Supplementary file1 (DOCX 22 KB)

## Data Availability

The datasets used and analysed during the current study are available from the corresponding author on reasonable request.
