# Supplementary material for: Clot lysis time and thrombin generation in patients undergoing transcatheter aortic valve implantation
Source: J Thromb Thrombolysis. 2024 Aug 8;58(1):50–61. doi: 10.1007/s11239-024-03027-5 (PMC11762420; doi:10.1007/s11239-024-03027-5)
Supplement: Supplementary file 1 — Supplementary file1 (DOCX 22 KB) [file 11239_2024_3027_MOESM1_ESM.docx]

**SUPPLEMENTARY MATERIALS**

**Table S1**. Correlations between clot lysis time, thrombin generation and echocardiographic values in patients before TAVI procedure.

|  | EF, % | | V max, m/s | | Gradient max, mmHg | | Gradient mean, mmHg | | AVA (VTI), cm2 | | AVAi, cm2/m2 | |
| --- | --- | --- | --- | --- | --- | --- | --- | --- | --- | --- | --- | --- |
|  | r | p-value | r | p-value | r | p-value | r | p-value | r | p-value | r | p-value |
| Fibrinogen, g/L | -0.09 | 0.53 | -0.02 | 0.91 | 0.10 | 0.48 | 0.13 | 0.33 | -0.20 | 0.20 | -0.20 | 0.20 |
| CLT, min | 0.13 | 0.31 | -0.03 | 0.87 | 0.09 | 0.49 | **0.32** | **0.01** | -0.07 | 0.62 | 0.004 | 0.98 |
| Lag time, min | 0.006 | 0.96 | -0.11 | 0.47 | **-0.27** | **0.04** | -0.15 | 0.25 | **-0.29** | **0.049** | -0.23 | 0.14 |
| ETP, nM x min | -0.18 | 0.17 | -0.10 | 0.51 | -0.0002 | 0.99 | 0.002 | 0.99 | 0.04 | 0.81 | 0.12 | 0.44 |
| Peak, nM | -0.12 | 0.35 | 0.06 | 0.68 | 0.25 | 0.06 | 0.20 | 0.12 | **0.38** | **0.008** | 0.30 | 0.06 |
| ttPeak, min | 0.057 | 0.66 | -0.12 | 0.44 | **-0.32** | **0.01** | -0.22 | 0.09 | **-0.34** | **0.02** | -0.27 | 0.08 |

**Captions to Table S1**. Data shown as spearman correlation coefficient (r) and p-value. **Abbreviations**: AVA, aortic valve area; AVAi; indexed aortic valve area per body surface area; EF, left ventricular ejection fraction; Gradient max, maximal gradient through aortic valve; Gradient mean, mean gradient through aortic valve; V max, maximal velocity through aortic valve; others see Table 2.

**Table S2**. Correlations between clot lysis time, thrombin generation and echocardiographic values in patients after TAVI procedure.

|  | EF [%] | | V max [m/s] | | Gradient max | | Gradient mean | | AVA (VTI) | | AVAi | |
| --- | --- | --- | --- | --- | --- | --- | --- | --- | --- | --- | --- | --- |
|  | r | p-value | r | p-value | r | p-value | r | p-value | r | p-value | r | p-value |
| Fibrinogen, mg/dL | -0.16 | 0.39 | 0.06 | 0.78 | -0.09 | 0.45 | 0.05 | 0.79 | 0.13 | 0.53 | 0.04 | 0.85 |
| CLT, min | 0.10 | 0.54 | 0.24 | 0.21 | 0.16 | 0.33 | 0.09 | 0.57 | 0.04 | 0.85 | -0.21 | 0.29 |
| Lag time, min | 0.12 | 0.43 | 0.09 | 0.64 | 0.20 | 0.22 | 0.18 | 0.26 | 0.09 | 0.65 | 0.03 | 0.90 |
| ETP, nM x min | -0.24 | 0.12 | -0.006 | 0.98 | -0.02 | 0.91 | -0.041 | 0.80 | -0.003 | 0.99 | 0.22 | 0.28 |
| Peak, nM | -0.29 | 0.06 | 0.10 | 0.62 | -0.08 | 0.64 | -0.15 | 0.33 | 0.07 | 0.71 | 0.05 | 0.82 |
| ttPeak, min | 0.13 | 0.41 | -0.009 | 0.96 | 0.14 | 0.38 | 0.10 | 0.53 | 0.26 | 0.17 | 0.19 | 0.33 |

**Captions to Table S2**. Data shown as spearman correlation coefficient (r) and p-value.

Abbreviations: see Table S1.

**Table S3**. Results of univariable and multivariable analysis including clinical characteristics and baseline fibrin clot properties to predict a decrease (delta) in mean aortic flow gradient, representing hemodynamic success of TAVI.

|  | **Univariable analysis** | **Multivariable analysis** |
| --- | --- | --- |
| **Baseline characteristics** | | |
| Age (years) | 0.943 |  |
| Gender, male | 0.097 |  |
| BMI [kg/m2] | 0.398 |  |
| **Medical history and concomitant diseases** | |  |
| NYHA class |  |  |
| EuroSCORE II [%] | 0.767 |  |
| Prior myocardial infarction | **0.051** | 0.925 |
| Prior PCI | 0.147 |  |
| Prior CABG | 0.449 |  |
| Atrial fibrillation | 0.817 |  |
| Prior stroke/TIA | 0.733 |  |
| CKD > G3a | 0.220 |  |
| Diabetes mellitus | 0.651 |  |
| Hypertension | 0.270 |  |
| COPD | 0.479 |  |
| **Laboratory data** | |  |
| Hemoglobin [g/dL] | **0.074** | 0.407 |
| Leukocytes [G/l] | 0.426 |  |
| Platelets [G/l] | 0.113 |  |
| Estimated GFR [ml/min/1.73 m^2^] | 0.751 |  |
| NT – proBNP [ng/l] | 0.933 |  |
| CRP [mg/l] | **<0.001** | 0.003 |
| **Echocardiography before TAVI** | |  |
| Ejection fraction [%] | 0.129 |  |
| V max [m/s] | **0.003** | 0.301 |
| AVA (VTI) | 0.512 |  |
| AVAi | 0.905 |  |
| **Clot lysis and thrombin generation before TAVI** | |  |
| CLT (min) | **0.02** | 0.02 |
| Lag time (min) | 0.679 |  |
| ETP (nM x min) | 0.575 |  |
| Peak (nM) | 0.589 |  |
| Time to peak (min) | 0.307 |  |
| Fibrinogen (mg/dL) | 0.216 |  |

Captions to Table S3. Data shown as p-value for univariable and multivariable analysis. Variables predictive for delta mean gradient in univariable analysis at p<0.1 were taken into account in multivariable analysis. Abbreviations see Table S1.
